# Supplementary material for: Perceptual decisions interfere more with eye movements than with reach movements
Source: Commun Biol. 2023 Aug 30;6:882. doi: 10.1038/s42003-023-05249-4 (PMC10468498; doi:10.1038/s42003-023-05249-4)
Supplement: Supplementary file 2 — Supplementary Information [file 42003_2023_5249_MOESM2_ESM.pdf]

Supplementary Information

Perceptual decisions interfere more with eye movements than with reach movements

Kazumichi Matsumiya & Shota Furukawa

Graduate School of Information Sciences, Tohoku University, Sendai, Japan

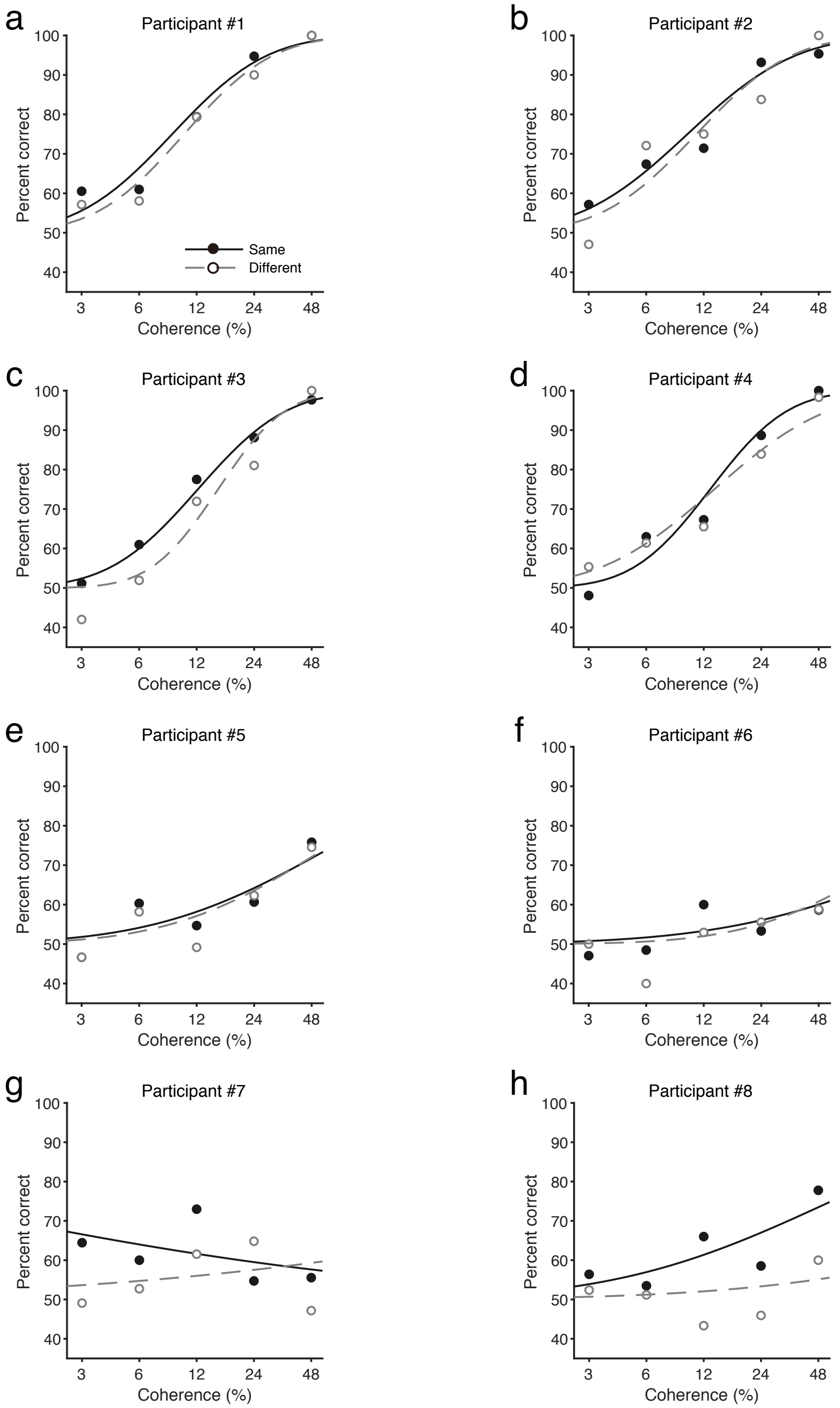

Supplementary Figure 1. Motion direction discrimination accuracy as a function of motion coherence for the active decision-making condition when simultaneous judgement-irrelevant saccades and reaches are made. Participants actively discriminated the direction of the motion stimuli and were then instructed to make simultaneous saccade and reach movements to either the same target (solid symbols) or different targets (open symbols). Cumulative Gaussian functions were fit to the data of each condition with least squares regression. (**a**–**h**) Each panel represents a different participant.

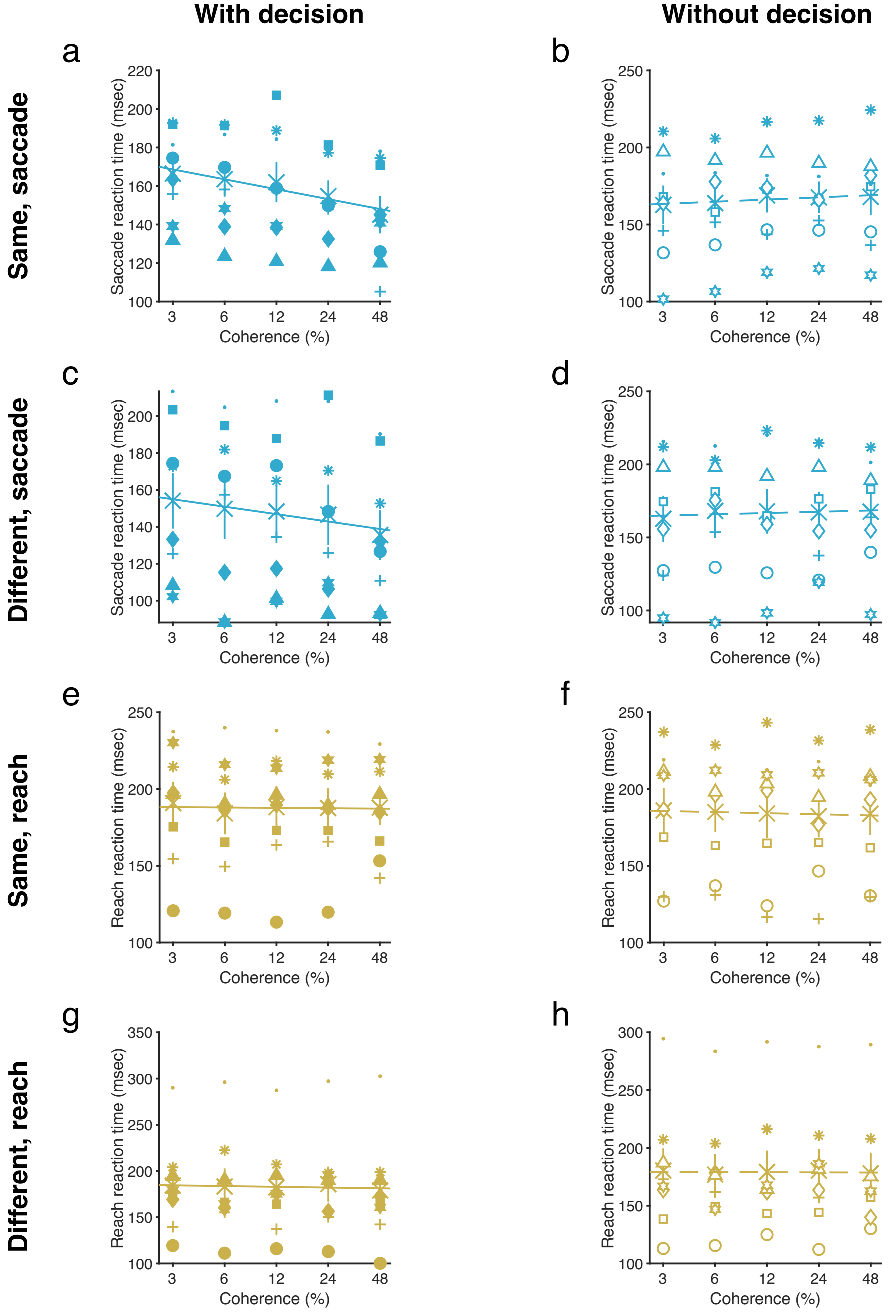

Supplementary Figure 2. Reaction times of simultaneous judgement-irrelevant saccades and reaches. Saccade reaction times for the same (**a**, **b**) and different (**c**, **d**) tasks as a function of motion coherence. Reach reaction times for the same (**e**, **f**) and different (**g**, **h**) tasks as a function of motion coherence. Solid and open symbols represent the active decision-making and passive viewing conditions, respectively. Different symbols other than the cross symbol represent different participants. Each cross symbol represents the average value for each motion coherence. Solid and dashed lines are the fitted lines for the active decision-making and passive viewing conditions, respectively. Crosses represent the mean ± standard error (n = 8). Error bars represent standard errors.

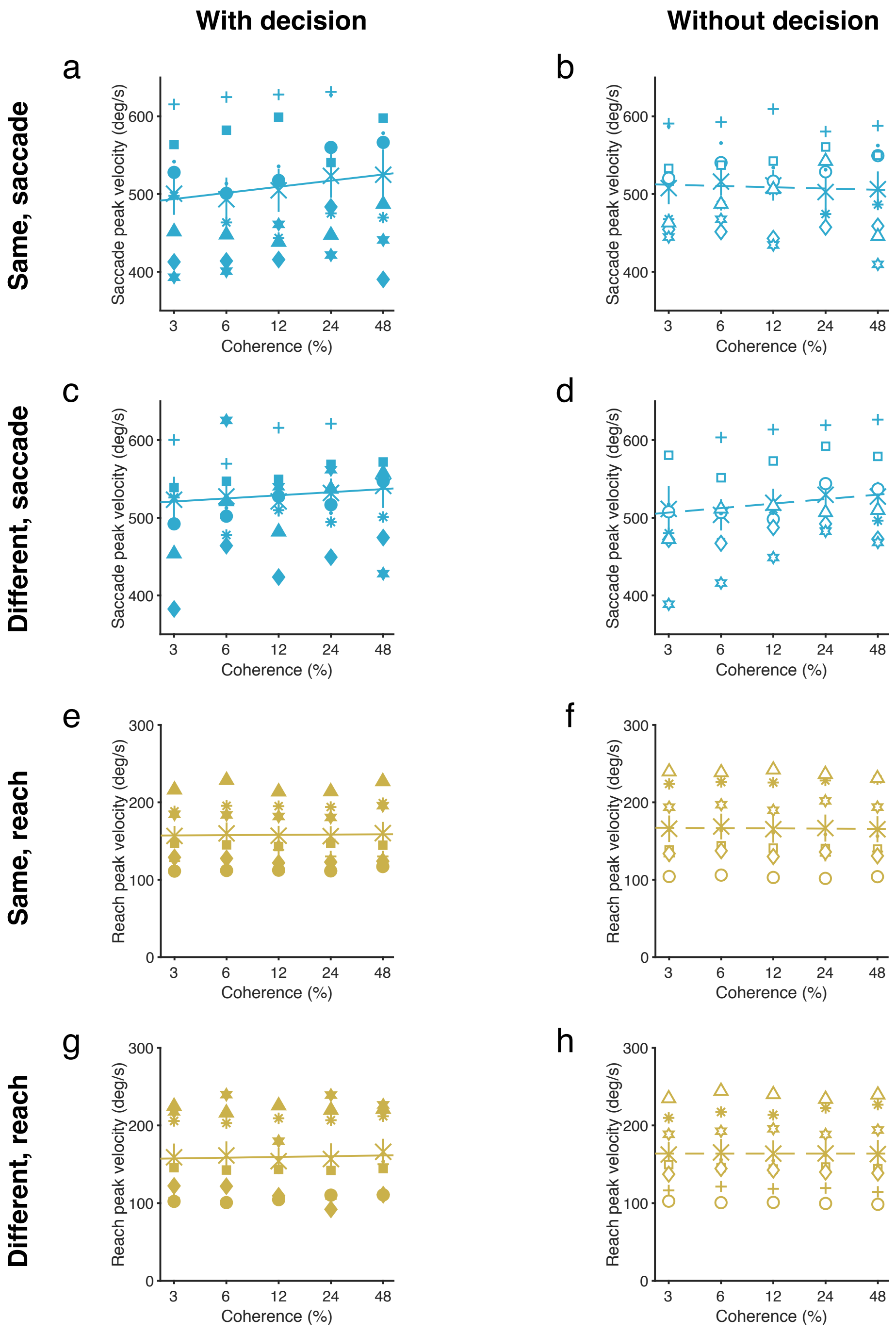

Supplementary Figure 3. Peak velocities of simultaneous judgement-irrelevant saccades and reaches. Saccade peak velocities for the same (**a**, **b**) and different (**c**, **d**) tasks as a function of motion coherence. Reach peak velocities for the same (**e**, **f**) and different (**g**, **h**) tasks as a function of motion coherence. Solid and open symbols represent the active decision-making and passive viewing conditions, respectively. Different symbols other than the cross symbol represent different participants. Each cross symbol represents the average value for each motion coherence. Solid and dashed lines are the fitted lines for the active decision-making and passive viewing conditions, respectively. Crosses represent the mean ± standard error (n = 8). Error bars represent standard errors.

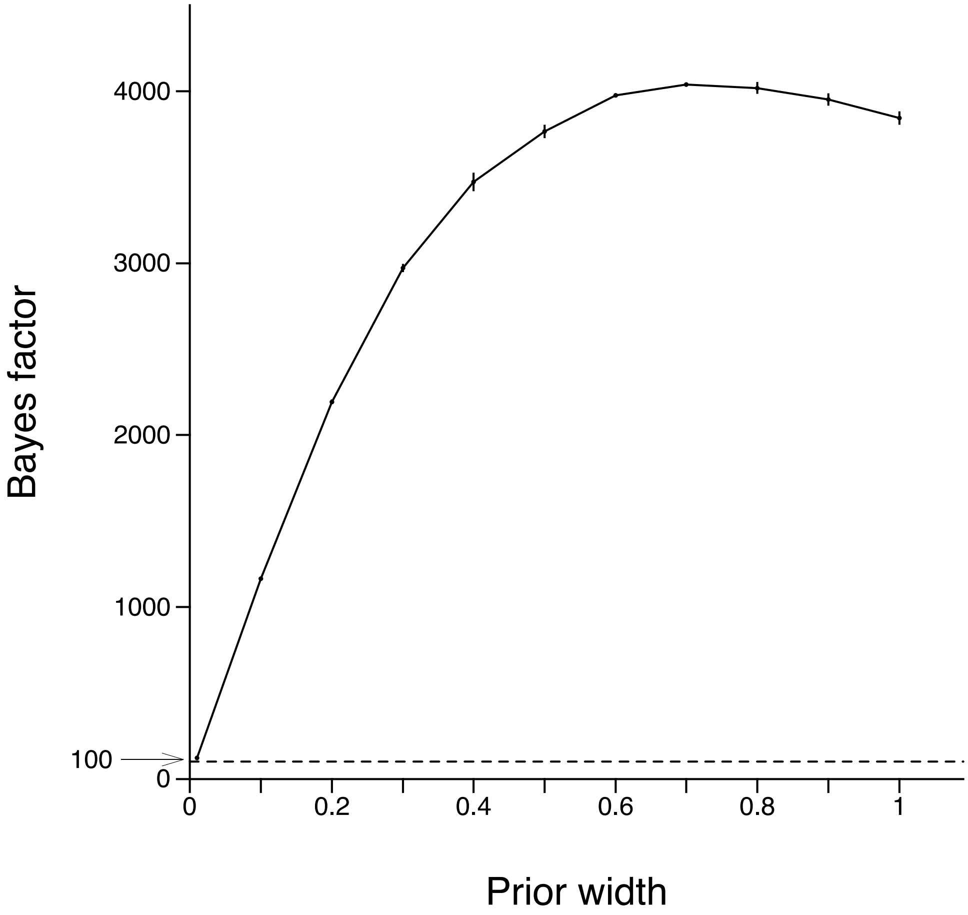

Supplementary Figure 4. Bayes factor (*BF*) as a function of the width of the prior for reach peak velocity. The *BF* value is the average of 3 iterations of the Markov chain Monte Carlo method with a maximum of 10,000 estimates. The dotted line represents a *BF* value of 100. To analyze how the *BF* changes as a function of the width of the prior, we varied the width of the prior from 0.01 to 1. As a result, the *BF* value decreased significantly when the width of the prior was less than 0.4. However, even though the width of the prior was set to 0.01, the *BF* was still 120.05. This suggests that the disagreement between *p*-value and *BF* for reach peak velocities holds for a wide range of the width of the prior from 0.01 to 1. Results are the mean ± standard error. Error bars represent standard errors.

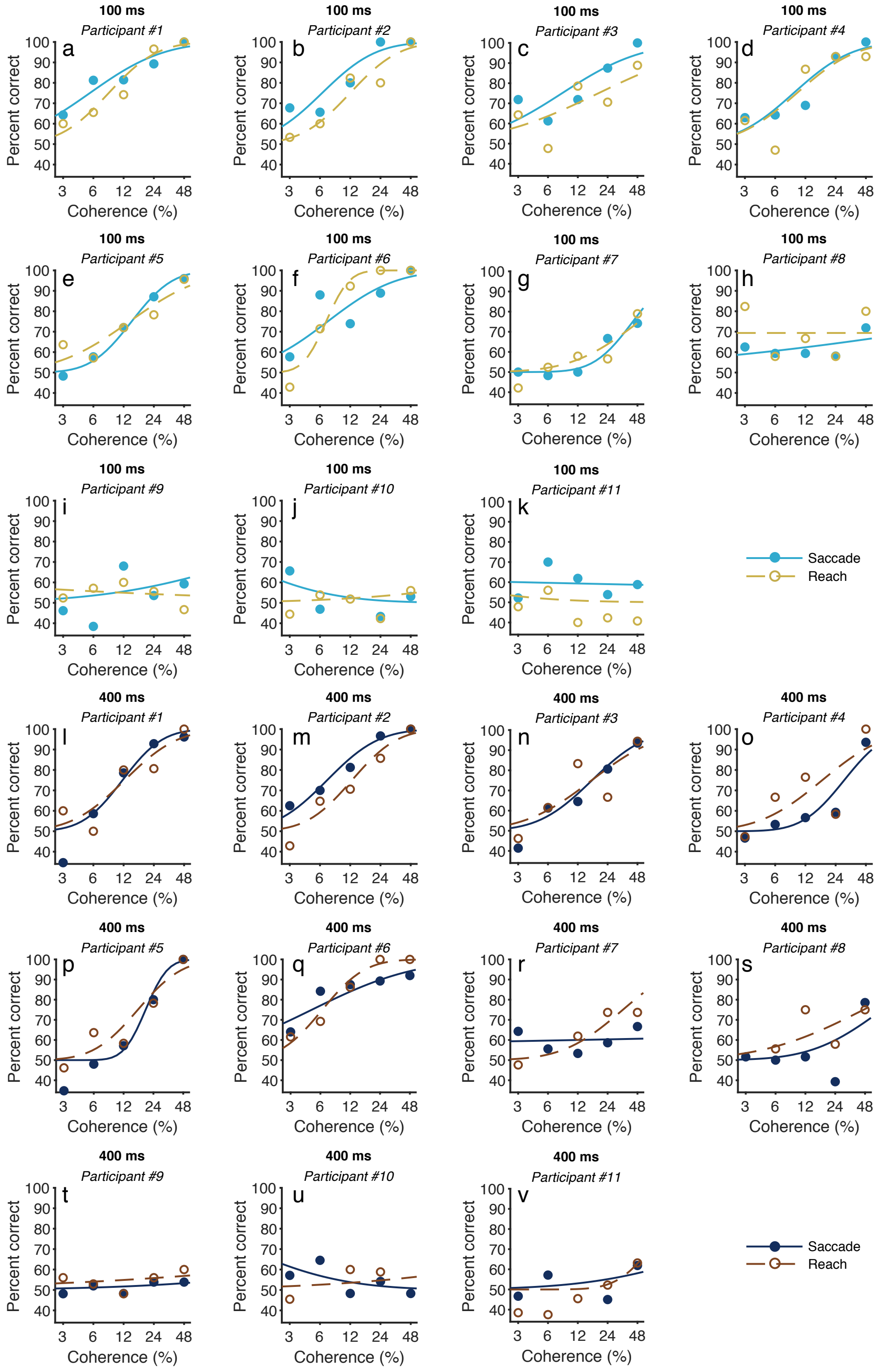

Supplementary Figure 5. Motion direction discrimination accuracy as a function of motion coherence in the saccade-only and reach-only tasks. Participants actively discriminated the direction of the motion stimuli and were then instructed to make a saccade without a reach movement to the decision-irrelevant target (solid symbols) or to make a reach without a saccade movement to the decision-irrelevant target (open symbols). (**a–k**) 100-ms duration. (**l–v**) 400-ms duration. Cumulative Gaussian functions were fit to the mean data of each condition with least squares regression. Each panel for each duration condition represents a different participant.

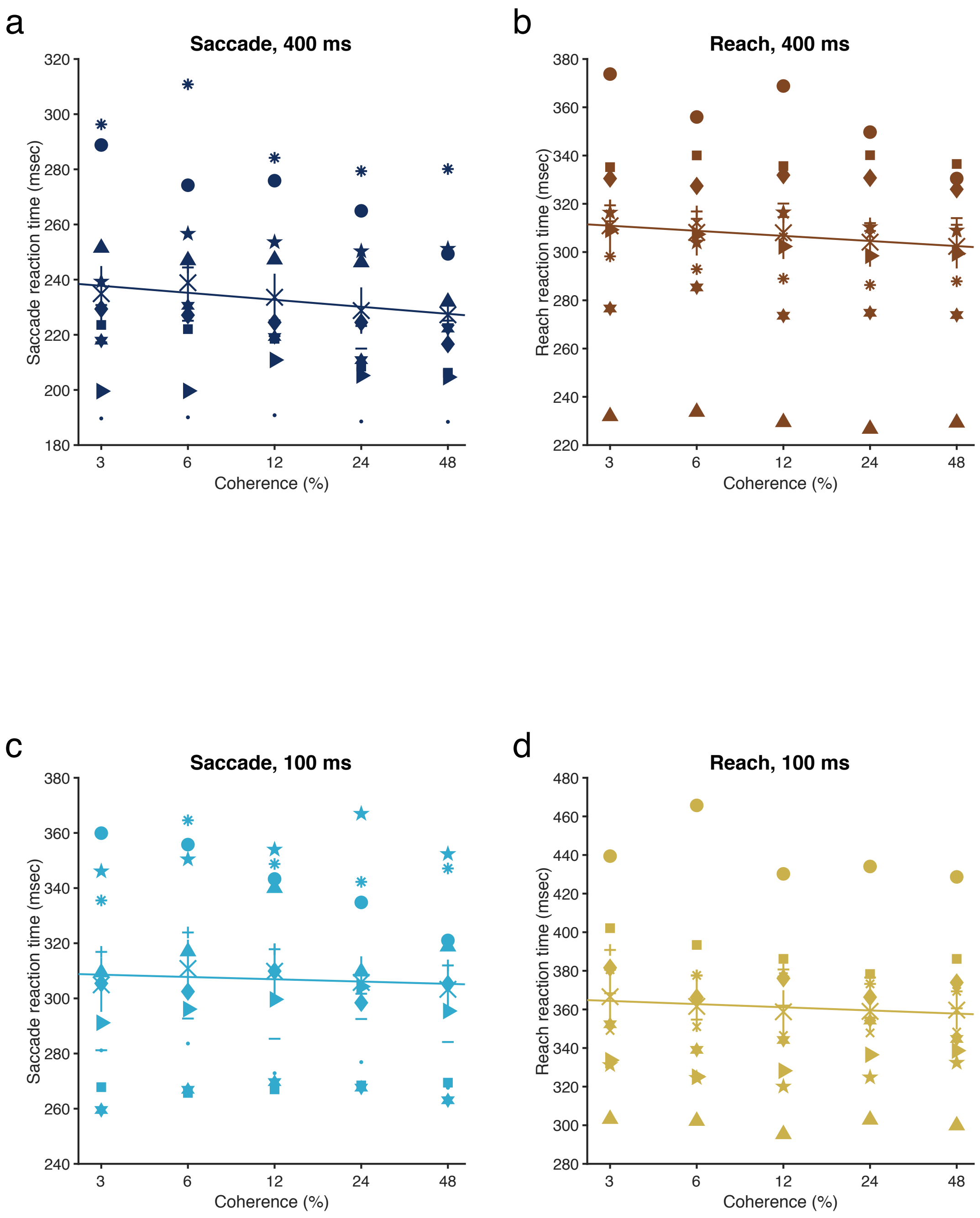

Supplementary Figure 6. Reaction times for saccades and reaches as a function of motion coherence in the saccade-only and reach-only tasks. Saccade reaction times for the long (400 ms; **a**) and short (100 ms; **c**) duration conditions as a function of motion coherence. Reach reaction times for the long (400 ms; **b**) and long (100 ms; **d**) duration conditions as a function of motion coherence. Different symbols other than the cross symbol represent different participants. Each cross symbol represents the average value for each motion coherence. Solid lines are the fitted lines. Crosses represent the mean ± standard error (n = 11). Error bars represent standard errors.

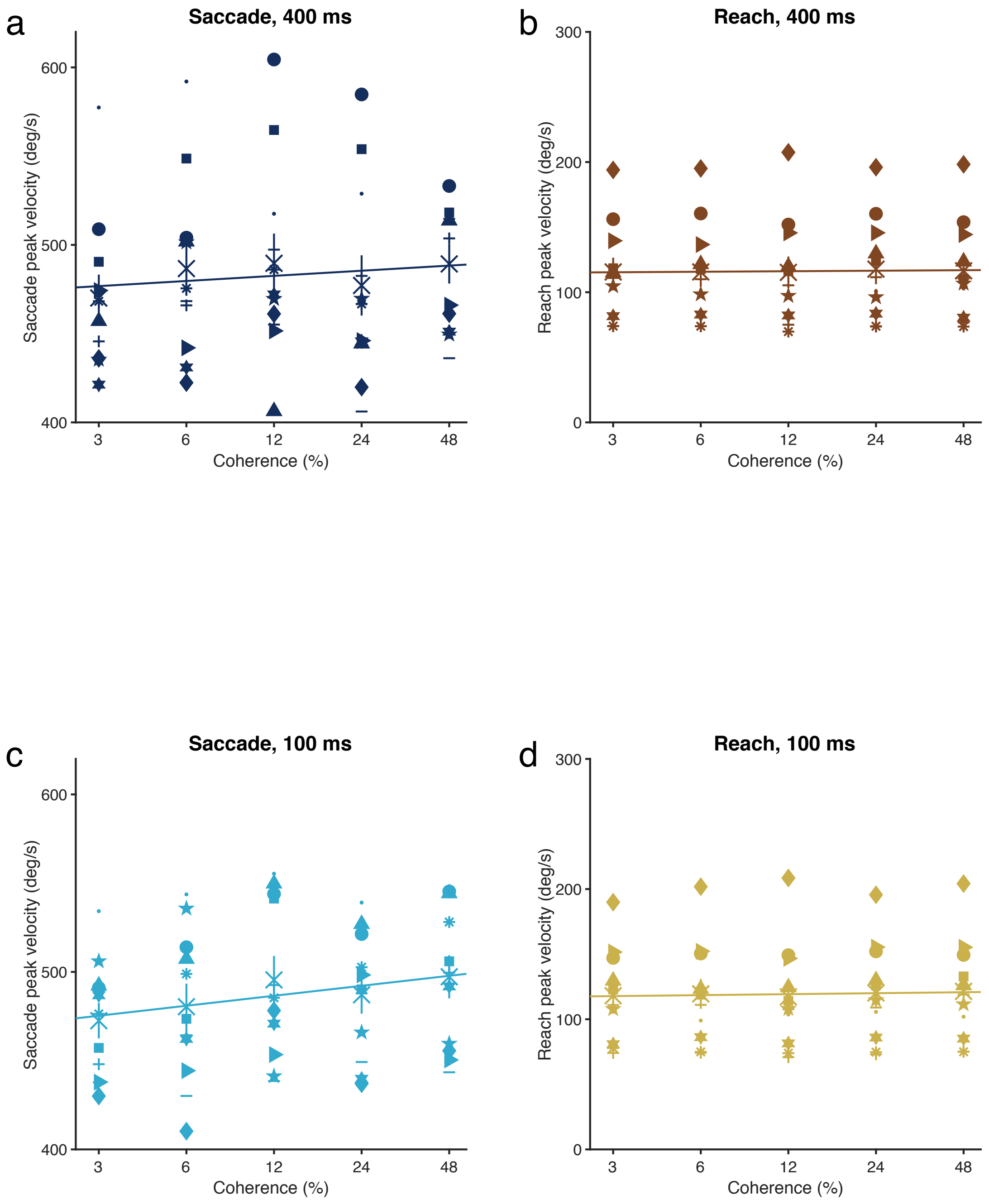

Supplementary Figure 7. Peak velocities for saccades and reaches as a function of motion coherence in the saccade-only and reach-only tasks. Saccade peak velocities for the long (400 ms; **a**) and short (100 ms; **c**) duration conditions as a function of motion coherence. Reach peak velocities for the long (400 ms; **b**) and short (100 ms; **d**) duration conditions as a function of motion coherence. Different symbols other than the cross symbol represent different participants. Each cross symbol represents the average value for each motion coherence. Solid lines are the fitted lines. Crosses represent the mean ± standard error (n = 11). Error bars represent standard errors.

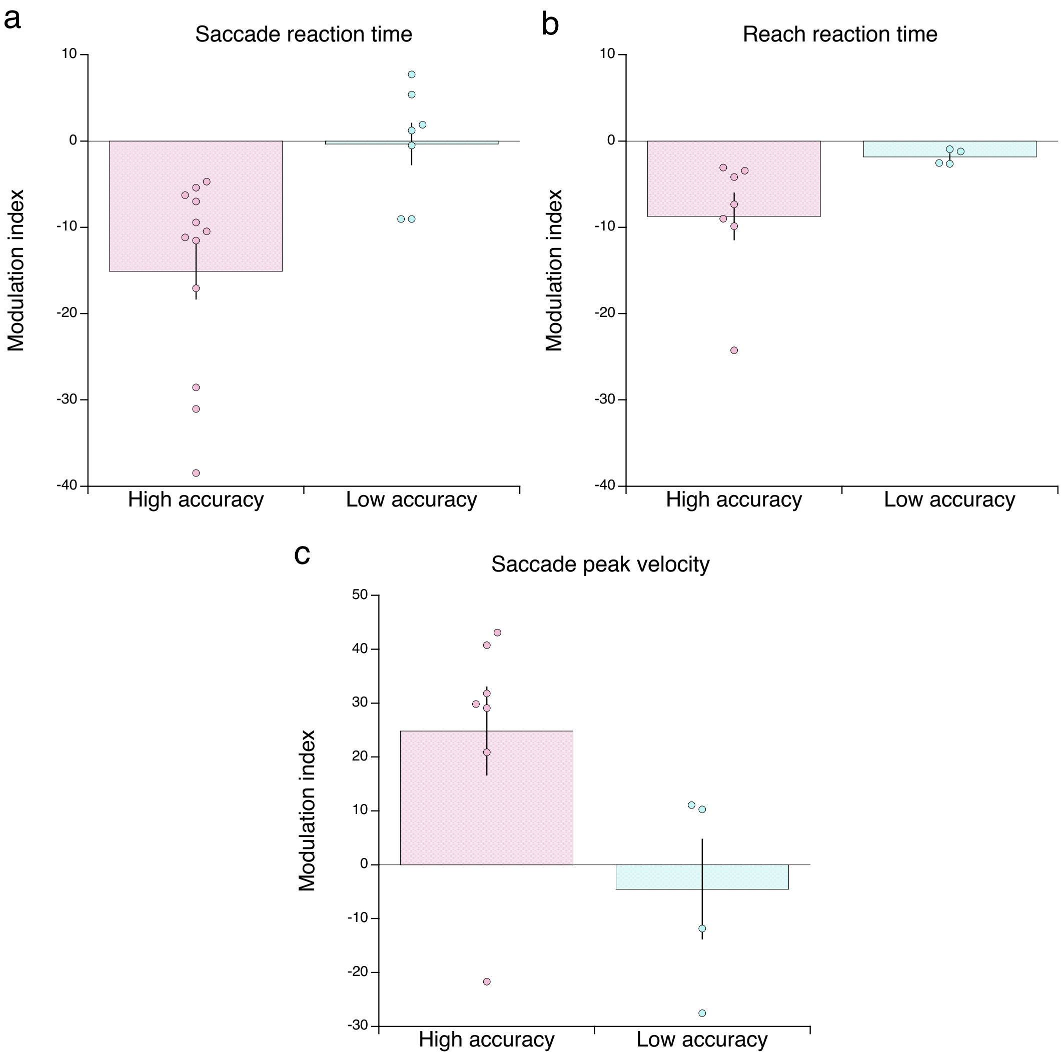

Supplementary Figure 8. Degree of modulation of reaction time and velocity by motion coherence for high- and low-accuracy groups. (**a**) Saccade reaction time. (**b**) Reach reaction time. (**c**) Saccade peak velocity. The saccade reaction time data were collected from experiments 1 and 2. The reach reaction time data were collected from experiment 2. The saccade velocity data were collected from experiment 2. For the saccade movement data in experiment 1, saccade reaction times for the same and different conditions were averaged for each motion coherence. For each of the saccade and reach movement data in experiment 2, reaction times for the short- and long-duration conditions were averaged for each motion coherence. Saccade peak velocities for the short- and long-duration conditions were averaged for each motion coherence. The degree of modulation of reaction time and velocity by motion coherence (the modulation index) was calculated as the slope of the reaction time and velocity against motion coherence, respectively. A positive value of the modulation index represents an increase in motor performance with motion coherence. The modulation indices were classified into high-accuracy and low-accuracy groups. The high-accuracy group consisted of participants with 75% or more perceptual accuracy at the highest motion coherence level. Each circle symbol represents a different participant. Bars represent the mean ± standard error (saccade reaction times: n = 12 for the high-accuracy group; n = 7 for the low-accuracy group; reach reaction times and saccade peak velocities: n = 7 for the high-accuracy group; n = 4 for the low-accuracy group). Error bars represent standard errors. For statistical evaluation, a t-test of the group mean data was performed.

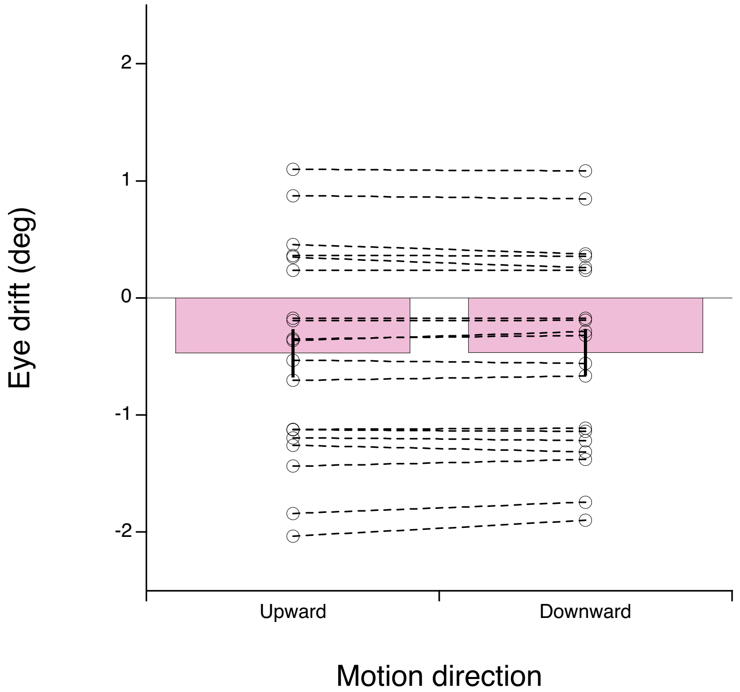

Supplementary Figure 9. Stability of participants' fixation during the presentation of motion stimuli. Bars represent the mean ± standard error. Error bars represent standard errors. Circle symbols and dotted lines represent different participants. A positive value of the drift represents the upward direction of the drift. In this study, participants were instructed to keep their gaze on the fixation point during the presentation of the motion stimulus. The fixation point was presented at the centre of the motion stimulus. The direction of motion was up or down. The viewing durations of the motion stimulus were 100 ms in experiment 1 and 100 or 400 ms in experiment 2. We analysed how much the eyes drifted during the presentation of the motion stimulus for experiments 1 and 2. If the motion stimulus elicits strong eye drift, the direction of the eye drift should greatly change depending on the direction of the motion stimulus. There was no significant difference in eye drift between the upward and downward motion directions ($t_{18} = -0.45$, $P = 0.66$, $d = 0.0066$). These results indicate that the participants' fixation was relatively stable during the presentation of the motion stimulus. For statistical evaluation, a paired *t*-test of the group mean data was performed. $n = 19$.
