# Supplementary material for: Perceptual decisions interfere more with eye movements than with reach movements
Source: Commun Biol. 2023 Aug 30;6:882. doi: 10.1038/s42003-023-05249-4 (PMC10468498; doi:10.1038/s42003-023-05249-4)
Supplement: Supplementary file 3 — Reporting Summary [file 42003_2023_5249_MOESM3_ESM.pdf]

Corresponding author(s): Kazumichi Matsumiya

Last updated by author(s): 2023/8/12

Reporting Summary

Nature Portfolio wishes to improve the reproducibility of the work that we publish. This form provides structure for consistency and transparency in reporting. For further information on Nature Portfolio policies, see our Editorial Policies and the Editorial Policy Checklist.

Statistics

For all statistical analyses, confirm that the following items are present in the figure legend, table legend, main text, or Methods section.

n/a	Confirmed	
☐	☒	The exact sample size (*n*) for each experimental group/condition, given as a discrete number and unit of measurement
☐	☒	A statement on whether measurements were taken from distinct samples or whether the same sample was measured repeatedly
☐	☒	The statistical test(s) used AND whether they are one- or two-sided<br>*Only common tests should be described solely by name; describe more complex techniques in the Methods section.*
☐	☒	A description of all covariates tested
☐	☒	A description of any assumptions or corrections, such as tests of normality and adjustment for multiple comparisons
☐	☒	A full description of the statistical parameters including central tendency (e.g. means) or other basic estimates (e.g. regression coefficient) AND variation (e.g. standard deviation) or associated estimates of uncertainty (e.g. confidence intervals)
☐	☒	For null hypothesis testing, the test statistic (e.g. *F*, *t*, *r*) with confidence intervals, effect sizes, degrees of freedom and *P* value noted<br>*Give P values as exact values whenever suitable.*
☒	☐	For Bayesian analysis, information on the choice of priors and Markov chain Monte Carlo settings
☒	☐	For hierarchical and complex designs, identification of the appropriate level for tests and full reporting of outcomes
☐	☒	Estimates of effect sizes (e.g. Cohen's *d*, Pearson's *r*), indicating how they were calculated

Our web collection on statistics for biologists contains articles on many of the points above.

Software and code

Policy information about availability of computer code

Data collection	Worldviz Vizard 6 was used to collect data.
Data analysis	MATLAB R2021a was used for data preprocessing. SPSS ver 25 was used for statistical analysis. R package BayesFactor 0.9.12-4.4 was used for Bayes factor analysis.

For manuscripts utilizing custom algorithms or software that are central to the research but not yet described in published literature, software must be made available to editors and reviewers. We strongly encourage code deposition in a community repository (e.g. GitHub). See the Nature Portfolio guidelines for submitting code & software for further information.

Data

Policy information about availability of data

All manuscripts must include a data availability statement. This statement should provide the following information, where applicable:

- Accession codes, unique identifiers, or web links for publicly available datasets
- A description of any restrictions on data availability
- For clinical datasets or third party data, please ensure that the statement adheres to our policy

The data supporting our study are available at an OSF repository (https://osf.io/vq95c/).

Human research participants

Policy information about studies involving human research participants and Sex and Gender in Research.

Reporting on sex and gender	Nineteen participants (5 women; mean age = 23.05 years) were recruited for the current study.
Population characteristics	Participants were undergraduate and graduate students of Tohoku University in Japan. All participants had normal or corrected-to-normal vision.
Recruitment	Participants were recruited from a paid participant pool established in Tohoku University.
Ethics oversight	This study was approved by the Ethics Committee of the Graduate School of Information Sciences, Tohoku University.

Note that full information on the approval of the study protocol must also be provided in the manuscript.

Field-specific reporting

Please select the one below that is the best fit for your research. If you are not sure, read the appropriate sections before making your selection.

☒ Life sciences ☐ Behavioural & social sciences ☐ Ecological, evolutionary & environmental sciences

For a reference copy of the document with all sections, see nature.com/documents/nr-reporting-summary-flat.pdf

Life sciences study design

All studies must disclose on these points even when the disclosure is negative.

Sample size	A total of 19 subjects were participated in this study. This sample size is typical for human psychophysical experiments.
Data exclusions	We excluded trials in which participants failed to make the movements of the eye or hand as instructed and trials in which the first saccade or reach amplitude was within 5 degrees (21.5% of total trials).
Replication	We used the methods developed by a previous study. We replicated the same results as the previous study in a control condition.
Randomization	Properties of saccade or reach targets were determined using a random-number generator.
Blinding	Participants were not divided into different groups. Our experiments were simply designed, because participants were asked to look at some visual stimuli and their eye and hand movements were measured.

Reporting for specific materials, systems and methods

We require information from authors about some types of materials, experimental systems and methods used in many studies. Here, indicate whether each material, system or method listed is relevant to your study. If you are not sure if a list item applies to your research, read the appropriate section before selecting a response.

Materials & experimental systems

n/a	Involved in the study	
☒	☐	Antibodies
☒	☐	Eukaryotic cell lines
☒	☐	Palaeontology and archaeology
☒	☐	Animals and other organisms
☒	☐	Clinical data
☒	☐	Dual use research of concern

Methods

n/a	Involved in the study	
☒	☐	ChIP-seq
☒	☐	Flow cytometry
☒	☐	MRI-based neuroimaging
